# Auditory Memory deficit in Elderly People with Hearing Loss 

**Published:** 2013-06

**Authors:** Zahra Shahidipour, Ahmad Geshani, Zahra Jafari, Shohreh Jalaie, Elham Khosravifard

**Affiliations:** 1 *Department of Audiology, Faculty of Rehabilitation, Tehran University of Medical Sciences, Tehran, Iran.*; 2 *Rehabilitation Research Center, Faculty of Rehabilitation, Tehran University of Medical Sciences, Tehran, Iran.*; 3 *Department of Statistic, Faculty of Management, Tehran University of Medical Sciences, Tehran, Iran.*; 4 *Department of Audiology, Faculty of Rehabilitation, Shahid Beheshti University of Medical Sciences, Tehran Iran.*

**Keywords:** Cognition, Dichotic verbal Memory Test, Hearing loss, Memory disorders, Presbycusis

## Abstract

**Introduction:**

Hearing loss is one of the most common problems in elderly people. Functional side effects of hearing loss are various. Due to the fact that hearing loss is the common impairment in elderly people; the importance of its possible effects on auditory memory is undeniable. This study aims to focus on the hearing loss effects on auditory memory.

**Materials and Methods:**

Dichotic Auditory Memory Test (DVMT) was performed on 47 elderly people, aged 60 to 80; that were divided in two groups, the first group consisted of elderly people with hearing range of 24 normal and the second one consisted of 23 elderly people with bilateral symmetrical ranged from mild to moderate Sensorineural hearing loss in the high frequency due to aging in both genders.

**Results:**

Significant difference was observed in DVMT between elderly people with normal hearing and those with hearing loss (P<0.22). According to the correlation test between Pure Tone Average (PTA) and the mean score of DVMT, increasing PTA resulted in reduction of DVMT score and this result was seen in both genders and all of the studied subjects.

**Conclusion:**

Apart from aging, age-related hearing loss has shown significant effect on auditory verbal memory. This result depicts the importance of auditory intervention to make better communicational skills and therefore auditory memory in this population.

## Introduction

Hearing loss is one of the most common problems in elderly people. It is the third chronic disease in elderly people after arthritis and hypertension. Based on the United States statistics, up to 40 to 45 % of elderly people older than 60 years old experience some degrees of hearing loss, this range increases to 83%for more than 70 years old people (1,2), and it is predicted that in 2030, 44 million people in the United State of America have some degrees of hearing loss (3). Age-related hearing loss, or presbycusis, is a progressive, bilateral and symmetric hearing deficit, primarily in high frequencies. The presbycusis reasons are not completely understood. There is a general consensus that presbycusis is the result of various types of physiological degeneration plus the accumulated effects of noise exposure, medical disorders and their treatment, as well as hereditary susceptibility (4). Typical degree of hearing loss in elderly population is mild to moderate, and in most cases, people face difficulties in speech perception )^5^-^7^). Moreover, presbycusis can lead to adverse effects on the physical, emotional, behavioral, social function and reduction in quality of life of older adults and particularly it effects on cognitive function (8-11). Cognitive function includes emotion, understanding, memory, learning, thinking, meditating, attention and logical functions. Based on many studies, there is an association between hearing loss and cognitive impairment (12-14); and there are evidences that sensory functioning is a strong late-life predictor of individual differences in intellectual functioning (15). Based on the Baltimore Longitudinal Study of Aging, audiometric frequency of hearing loss has been found to be independently associated with incident all-cause dementia (16). Moreover some evidences show that decline in memory, typically precedes dementia, and verbal memory impairment is one of the strongest predictors for the development of dementia or Alzheimer's disease (17-19).Valentijn and colleagues in 2005 found the significant relationship between sensory and cognitive functions, the study showed the reduction of auditory acuity (dB loss) during a 6-year follow-up study that predicted a decline in visual-verbal learning performance as a cognitive function (20). On the other hand, some studies claimed no association between hearing loss and cognitive function (21). Despite many efforts which have been successfully providing some knowledge bout hearing loss side effects, we still know little about the interactions between peripheral auditory mechanisms and cognitive function. This study was designed to explore the relationship between hearing loss and cognition function. Nowadays there are several cognitive tests to evaluate cognitive function. The studies show that verbal tests evaluate more extensively the relationship between hearing loss and cognition (22). One aspect of cognitive function is auditory memory, also called verbal memory. Auditory memory means the recipient of verbal stimulation, processing and saving data and finally retrieval of auditory information (23). 

The dichotic Verbal Memory Test (DVMT) is one of the most valid tests for detecting auditory-verbal memory deficits and differences in memory function between the hemispheres (24). The high sensitivity of dichotic tests in diagnosis of auditory memory deficits and detection of interhemispheric function difference are confirmed in several studies (25-27). The Persian version of Dichotic Verbal Memory Test in 2011 was constructed and validated by Aghamollaei and colleagues. Persian version of DVMT has good content validity and can be used in detecting the auditory-memory deficits (25).Therefore, due to the high accuracy of this test, it was chosen. 

With regard to the growing geriatric population, high prevalence of hearing loss among this group and the intensive functional effects of hearing loss, it is critical to gain knowledge about the relationship between hearing loss and cognition, in particular, auditory verbal memory to manage old patients with hearing deficit. The knowledge and information about the relation between the hearing loss and memory could be developed by fundamental-application studies like this. And such tests could become more empirical in clinical evaluation via these kinds of studies which can lead to precise consultation and referral of patients with hearing and cognitive impairments; therefore, the elderly people and family members can be helped to understand the problem and provide the means to improve the patients’ quality of life and prevent them from social isolation and depression or dementia. Furthermore, this study can provide a normal value of Persian version of DVMT for aged people with normal hearing and mild to moderate hearing loss of elderly people. Also, in view of the fact that the studies about the hearing loss effects on cognition are minor and in Iran , this study was conducted to evaluate the effects of hearing loss on auditory memory on the elderlies between 60-80 years old.

## Materials and Methods

This cross-sectional study was performed on 47 old people aged 60 to 80 years old since 2012 January till 2012 June. The participants included 24 elderly people with normal peripheral hearing (PTA < 25dBHL) and 23 elderly people with mild to moderate symmetrical high frequency age- related hearing loss (55>PTA>25 dBHL). This study was approved by the ethics committee of Tehran University of Medical Sciences.

 The participants were sampled non-randomly among available elderly people. At first, we went to Omid Aging Institution in Tehran and found the participants; the entrance criteria include (1): Symmetrical hearing thresholds better than 55 dBHL in three frequencies of 1000-2000 and 4000 Hz (2). Normal word recognition score (WRS>90%) in both ears (3). Absence of any aural disease by otoscopy and tympanometry examinations (4). Normal acoustic reflexes in 500 -1000 and 2000 in both ears (4). Right handedness (5) Persian native speaker (6). Literacy level of 9 years and more (7). To eliminate the confounding effect of neurological abnormalities on the results, elderly people with history of head injury or brain surgery and use of psychological medications and Epilepsy based on the person’s expressions were excluded from study.

To insure the cognitive health, Persian version of mini-mental state examination (MMSE) was performed for all participants. MMSE is a brief questionnaire that is used to screen for cognitive impairment. The sensitivity and specificity of Persian version of MMSE for cognitive screening of old people are respectively 90 and 84% (in cut off 21) (28).

Pure-tone and speech audiometry were accomplished in a double-walled, sound-treated audiometric booth, using a clinical audiometer (Intracoustic AC40) with supra-aural headphone (Telephonics TDH-39P). Based on hearing thresholds in frequencies of 1000-2000 and 4000, the participants were divided in two groups, the first group included elderly people with normal hearing and the second group included the elderly people with presbycusis.

Persian version of DVMT was performed at the Most Comfortable Level (MCL) of hearing by using the two-channel audiometer with CD player compatibility (Interacoustic AC40) with supra-aural headphone (Telephonics TDH-39P). DVMT includes three different lists that each one includes 10 words. 

The words of each list were presented to right / left ear while the inverse of the same word synchronously presented to the other ear. 

At the end of each list, the patient was asked to remind and repeat the word regardless of the order in list. The correct answers were inscribed by the examiner, then the memory score was calculated for each list separately; average of three lists as ear score, average of two ears as total score of memory for each person. 

Kolmogorov-Smirnov test was used to define normal distribution of data. Independent t-test was conducted to compare the mean of the results, and correlation function was done to determine the relationship between PTA and DVMT. For statistical analysis, software Spss.17 was used based on 0.05 significant level.

## Results

By using Kolmogorov-Smirnov test, all values ​​of the test data showed normal distribution (P>0.05).

**Fig 1:**
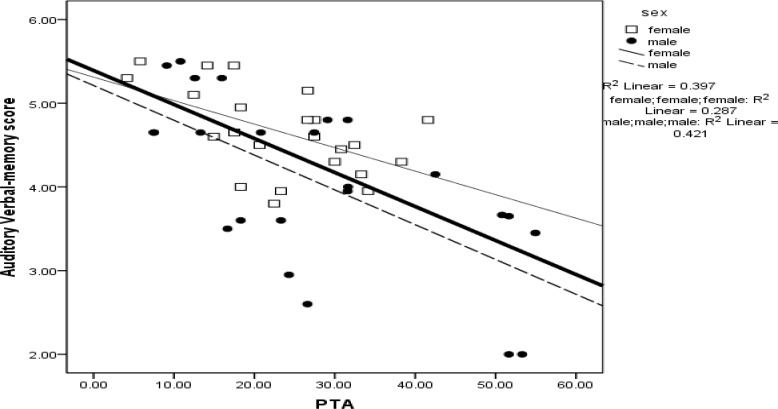
The Relationship between Pure Tone Average and Dichotic Auditory Verbal Memory Test. The mean score of DVMT was 4.65±.51 in females and 4.06±1 in males and 4.69±.87 in right ear and 4.01±.91 in left ear

In the current research, we studied hearing loss effect on DVMT. The average of DVMT score in aged people with normal hearing was 4.62 ±.75 and in aged people with hearing loss was 4.06 ±.86. Correlation between subjects PTA and DVMT score average is shown in figure 1. The horizontal axis represents participant PTA in dB HL and vertical axis represents scores of DVMT. 

## Discussion

Age-related hearing loss or presbycusis is one of the most common sensory deficits in the elderly, and as an increasingly important public health problem, it results in reduction in memory and quality of life (11). Moreover, it results in increasing the isolation, independence and frustration in both developed and developing counties. In this study, a comparison between DVMT results of elderly people with presbycusis and elderly people with normal hearing was made. Comparing these two groups depicts statistically significant differences between the scores (P= .022). The result showed the negative influence of hearing loss on DVMT. Although a sensory impairment seems to have independent impression, it can practically lead to processing in upper levels such as cognitive function.

Correlation between PTA and DVMT showed that increasing PTA resulted in the reduction of test score in all of the studied persons and in both genders separately. In fact more hearing loss causes more reduction in memory function.

Our result was consistent with Van Boxtel and colleagues in 2000 which studied the amount of immediate and delayed recall in an auditory verbal learning paradigm, their results indicated lower performance in subjects with mild to moderate hearing loss. And this result was observed even when the investigators make the situations clear in background noise and make improvement in signal and noise ratio (29). An association of auditory thresholds with memory was also found by Pearman and et al. who recruited 344 volunteers aged 55-93 years to participate in a nonverbal memory task. In this study, hearing impairment was associated with poor serial word-recall with regard to age and gender adjustment (22). Lin in 2011 conducted a study on 605 aged people with normal and impaired hearing, with the cognitive test concluded the digit symbol substitution test (DSST), a nonverbal test which evaluated the executive function and psychomotor processing. The results indicated that more hearing loss was significantly associated with lower scores on the DSST after adjustment for demographic factors and medical history (20). In contrast, some studies have not found similar associations, for example Gennis and colleagues in 1991 measured the cognition by two parts of the Wechsler Memory Scale and the Jacobs Cognitive Screening Test. Since this investigation was planned for a 5-year follow up, the results need the follow-up period of five years, so the study remains underpowered for conclusions (20). Also in another study by Zekveld in 2007 , it was indicated that hearing loss was not associated with decreased performance on the memory and attention tests and showed that the association of hearing loss with lower performance on cognitive tests that has been found in earlier studies seems nonexistent when nonverbal- visual tests of memory and attention were used )^30^ (. In this study, the nonverbal test was used for memory evaluation and the investigators emphasized that the lack of relationship was due to nature of nonverbal tests. And the other point was about the little sample size and also age distribution of subjects in the study, therefore the author indicated that the result couldn’t be generalized.

It is worth noting that the superiority of our study to previous research was audiometry evaluation based on available standard in vocal booth treatment with clinical audiometer. Although most studies utilized portable or screening audiometers or participants tested under varying environmental conditions , the effect of biased or imprecise assessments of hearing thresholds would likely decrease sensitivity to detect associations due to increased variance. The other superiority was utilizing a verbal test which can show the relationship between PTA and auditory memory test. 

There are different explanations for existence of hearing loss effect on cognitive function: first is the "common cause" hypothesis which is about brain aging and its adverse effect on many areas of the brain. This hypothesis states that auditory and cognitive declines are the characteristic of neural degeneration in many areas. The aging of the central nervous system may have a direct impact on many different brain functions (31). It should mention that in our study, we had a control group of elderly people with normal hearing to eliminate the aging effect; therefore, it can be possible to rule out the interference of common cause theory. A second explanation is about the information-processing model or degradation hypothesis, this hypothesis is more specific, and considers that poor auditory performance compromises cognitive performance by reducing the information available for processing (32). In our research, subjects with presbycusis had mild to moderate hearing loss and auditory verbal test was conducted at their MCL, therefore our results didn’t support Informational processing model. And third, the psychosocial factors such as depression and social isolation, may account for the variance in cognition in an aging population (33). Because our sample consisted of healthy community volunteers screened for cognition function by MMSE, and all the elderly people in this study should have score of more than 21, our findings do not support the psychological model of sensory loss and cognitive dysfunction. And finally, an Effortfullness hypothesis which means the extra efforts that a hearing impaired listener must expend to achieve perceptual success, may come at the cost of processing resources that might be available for encoding the speech content in memory (34). Results of our study support efforttfullness theory since it could refer to the lots of efforts for successful perception which impeded the subject’s ability to remind the word. Considering the complexities of the interaction between hearing and cognition to prove this theory, more study is needed.

The current study depicted another thought provoking result in which the DVMT scores were different between males and females. In many studies, it was observed that females performed better than males in immediate and delayed object recall and visual memory. In contrast males performed better in digit span, abstractive thought and spatial skills (35). In our study, females’ score was 4.65±.51 and males’ score was 4.06±1. As it is indicated that female’s mean scores are more than male’s scores. This result is consistent with Aghamollaei and colleagues who used DVMT (36). 

Female’s performance in verbal memory test could be due to the difference in brain structures between two groups. So far several studies showed the difference between structural and functional brain of males and females (37). Comparing the two ears, there were differences in test scores between left and right ears. This result is consistent with Aghamollaei and colleagues (38). The result of this study indicated “right ear advantage” in Persian version of dichotic auditory verbal memory test. The higher score of right ear is a result that was repeated several times in meaningful language information processing in dichotic presentation (39). On the basis of clinical evidence, it has been suggested that in 95.5% of the right-handed population, left hemisphere is specialized for speech perception thus the explanation of this finding was that, although there are both ipsilateral and contralateral neural pathways that connect the ears to the cerebral areas responsible for speech perception, during the performance of the dichotic listening task, the minor ipsilateral pathways are inhibited allowing the dominant contralateral pathways access to the areas specialized for speech perception and this phenomena is known as right ear advantage (40).

Our study also had some limitations as follows; The results were based on cross-sectional data instead of longitudinal study and variety of designs in cross-sectional study like using population- or selected samples, were not substituted for high-quality longitudinal data, and longitudinal study must be used to explore emergent hypotheses. Another limitation was the range of our subjects with hearing loss since the subjects had mild to moderate hearing loss. To investigate the hearing loss effect it also could be thought appropriate to evaluate old people with poorer hearing. 

## Conclusion

This research was conducted to study the relationship between sensory loss (i.e. hearing) and cognitive function (i.e. auditory memory). To investigate the hearing loss effect on auditory verbal memory, a kind of verbal memory test known as DVMT was used. The result showed that there were s significant relationship between hearing loss and auditory memory performance. Based on these results, it is suggested that the auditory-verbal memory assessments can be used as a supplementary tool for better management in clinical evaluation of geriatric patients; furthermore referral for auditory assessments is suggested in patients with cognitive impairment in psychological clinics. 
